# THE DAY-TO-DAY EXPERIENCES OF LATER-LIFE PROSOCIAL WORK: AN EXPERIENCE SAMPLING METHOD STUDY

**DOI:** 10.1093/geroni/igz038.1372

**Published:** 2019-11-08

**Authors:** Jeanne Nakamura, Dwight C K Tse, Ajit Mann, Laura Graham, Jordan Boeder, Kelsey Finley

**Affiliations:** Claremont Graduate University, Claremont, California, United States

## Abstract

As one form of productive aging, many older adults undertake significant prosocial activity. Alongside its contribution to the welfare of others, prosocial activity has been linked to a variety of positive outcomes for those undertaking it (e.g., higher life satisfaction). However, little is known about the impact of this activity on older adults’ day-to-day lives. We studied a national sample of about 150 older adults who give back to their communities on a regular basis in one of two ways: either by playing leadership roles in social-purpose organizations or as more traditional volunteer workers in such organizations. We employed the experience sampling method to describe and compare the experience of prosocial activity during a typical week for these two groups of highly engaged adults. While prosocial activity carries both costs and rewards, these show differences as well as similarities for the two groups. Implications for research and practice are discussed.

